# Electrochemical Recovery and Behaviors of Rare Earth (La, Ce, Pr, Nd, Sm, Eu, Gd, Tb, Dy, Ho, Er, Tm, and Yb) Ions on Ni Sheets

**DOI:** 10.3390/ma13235314

**Published:** 2020-11-24

**Authors:** Min Hee Joo, So Jeong Park, Sung Min Hong, Choong Kyun Rhee, Youngku Sohn

**Affiliations:** 1 Department of Chemistry, Chungnam National University, Daejeon 34134, Korea; wnalsgml4803@naver.com (M.H.J.); jsjs5921@naver.com (S.J.P.); qwqe212@naver.com (S.M.H.); ckrhee@cnu.ac.kr (C.K.R.); 2 Department of Chemical Engineering and Applied Chemistry, Chungnam National University, Daejeon 34134, Korea

**Keywords:** rare earth ions, electrochemical recovery, amperometry electrodeposition, cyclic voltammetry, actinide ions

## Abstract

The electrochemical behaviors of rare earth (RE) ions have extensively been studied because of their high potential applications to the reprocessing of used nuclear fuels and RE-containing materials. In the present study, we fully investigated the electrochemical behaviors of RE(III) (La, Ce, Pr, Nd, Sm, Eu, Gd, Tb, Dy, Ho, Er, Tm, and Yb) ions over a Ni sheet electrode in 0.1 M NaClO_4_ electrolyte solution by cyclic voltammetry between +0.5 and −1.5 V (vs. Ag/AgCl). Amperometry electrodeposition experiments were performed between −1.2 and −0.9 V to recover RE elements over the Ni sheet. The successfully RE-recovered Ni sheets were fully characterized by scanning electron microscopy, energy dispersive X-ray spectroscopy, Fourier transform infrared spectroscopy, X-ray photoelectron spectroscopy, and photoluminescence spectroscopy. The newly reported recovery data for RE(III) ions over a metal electrode provide valuable information on the development of the treatment methods of RE elements.

## 1. Introduction

Rare earth (RE) elements have widely been used in diverse industry materials such as magnets, fluorescent lamps, catalysts, and batteries [1,2,3,4,5]. Lanthanide (Ln) elements are commonly co-present with actinide (An) elements in nuclear oxide fuels. Therefore, the treatment and the recycling of used RE-containing industry materials and oxide fuels are of very attractive research projects [6,7,8,9,10]. In the reprocessing of spent nuclear oxide fuels, the pyroprocessing technology has been employed to reprocess Ln and An ions present in a pre-prepared electrolyte condition [11]. For this treatment, electrochemistry is an indispensable technique, and the understanding electrochemical behaviors (e.g., electrochemical reduction-oxidation reactions) of the ions over electrodes is very important. For the recovery of RE elements from used industry materials [12,13], the electrochemical recovery method has been a useful method to selectively recover a desired element in an electrolyte with mixed RE ions [12,13,14]. To achieve these goals, electrochemical behaviors of RE ions in an electrolyte have fundamentally been studied by electrochemistry [15,16,17,18,19]. Yang et al. studied electrochemical behaviors and electrodeposition of Eu (III) ions over a Al-Ga cathode, and co-reduction behaviors of Eu(III), Al(III) and Ga(III) over a W electrode [16]. They showed efficient reduction and co-reduction of Eu(III) ions over the electrodes. Liu et al. studied direct separation of U from RE (La, Nd, Ce, and Sm)-containing LiCl-KCl eutectic and showed that U was efficiently electrodeposited as Al-U alloys (Al_3_U and Al_4_U) on the Al electrode [17]. Kim and Lee focused on obtaining fundamental electrochemical properties of both Nd^3+/^Nd^2+^ and Ce^3+^/Ce in a molten LiCl-KCl eutectic salt at a high temperature of 773 K using a glassy carbon counter electrode, a Ag/Ag^+^ reference electrode, and a W working electrode, and reported the measured diffusion constant, the standard reduction potential, and the standard-state Gibb’s free energy of the RE ions [18]. Separation of a neighboring RE pair (e.g., Sm^3+/^Sm^2+^ and Eu^3+/^Eu^2+^) is another goal to be achieved. Ge et al. used a reactive Cu electrode for selective recovery as Cu-Sm intermetallic compounds [19]. Al-Eu compound was reported to be selectively recovered over an Al electrode at a negative potential of −2.225 V (vs. Ag/Ag^+^), higher than that of Al-Sm compound recovery on the electrode [19]. The molten-salt condition has commonly been performed at a high temperature [16,17,18,19] while the electrodeposition in ionic liquid conditions has been introduced for a mild temperature condition [20,21,22]. Xu et al. efficiently electrodeposited a rare earth iron alloy of Nd-Fe film on a Cu electrode in an ionic liquid condition containing Nd(III) and Fe(II) ions [20]. They proposed a deposition mechanism that Fe was initially deposited to subsequently catalyze the reduction of Nd(III) to Nd(0), and consequently Nd-Fe was co-deposited.

Although there are many literatures for the recovery of RE elements under various conditions, few studies have been systematically reported for the recovery of all the RE elements over a metal electrode in a NaClO_4_ condition at room temperature. Because the electrochemical behaviors of RE and An elements are very similar, the selective recovery and the treatment are challenging and needed to be continuously developed. The novelty of this study is that all the RE elements were introduced to show that they had a systematic relationship on redox potentials with atomic number. Therefore, this data set is very useful for better understanding the redox behaviors of rare earth ions and the development of Ln and An treatment methods as well as other heavy metal ions present in water.

## 2. Results and Discussion

**Figure 1:**
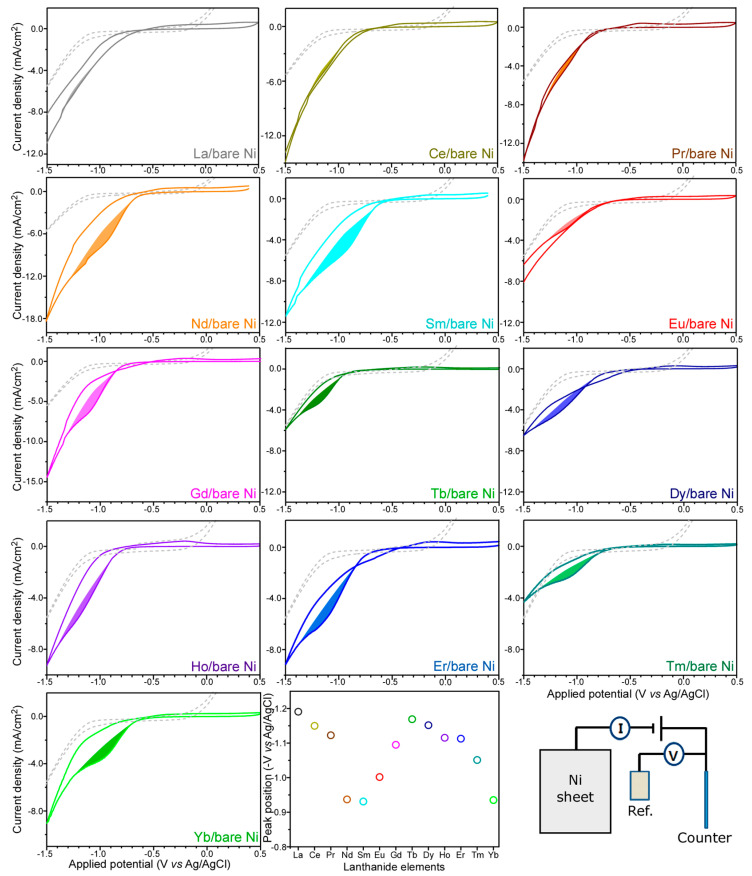
Cyclic voltammetry curves at a scan rate of 0.2 V/s for various 10 mM RE(III) ions in 0.1 M NaClO_4_ electrolyte over bare Ni sheets. The reduction peak positions (V vs. Ag/AgCl) are shown with RE elements. A schematic of the three-electrode system used in this study is shown.

To examine the electrochemical behaviors of RE(III) ions, Figure 1 displays cyclic voltammetry (CV) curves for various 10 mM RE(III) ions in 0.1 M NaClO_4_ electrolyte over bare Ni sheets. In a blank 0.1 M NaClO_4_ electrolyte, there was no discernible reduction–oxidation (redox) peaks except for the current increases below −1.0 V (vs. Ag/AgCl) and above 0.0 V. Upon addition of 10 mM RE(III) ions, a strong negative current increase was commonly observed, starting from −0.5 V. There was no significant current increase between −0.5 and +0.5 V. The negative current increase was due to both the hydrogen evolution reaction and the RE reduction/complexation. Interestingly, as the atomic number was increased (or as the atomic size was decreased) from #57 (La) to #70 (Yb), the reduction peak became distinctly appeared. This indicates that the reduction potentials for La (#57), Ce (#58), and Pr (#59) are close to the hydrogen reduction potential, and the reduction potentials for Er (#68), Tm (#69), and Yb (#70) are far from the hydrogen reduction potential. The reduction potential for La was barely detected at −1.2 V (vs. Ag/AgCl) and that for Yb was clearly seen at −0.95 V (vs. Ag/AgCl). The reduction potentials for Nd, Sm, and Eu were observed to be between −1.11 and −0.92 V (vs. Ag/AgCl). Xu et al. reported that the equilibrium potentials of RE elements were in a relationship with atomic size [23]. Herein, the present redox potentials of RE(III) ions appear to be also influenced by other factors such as electronegativity and complex formation degree.

**Figure 2:**
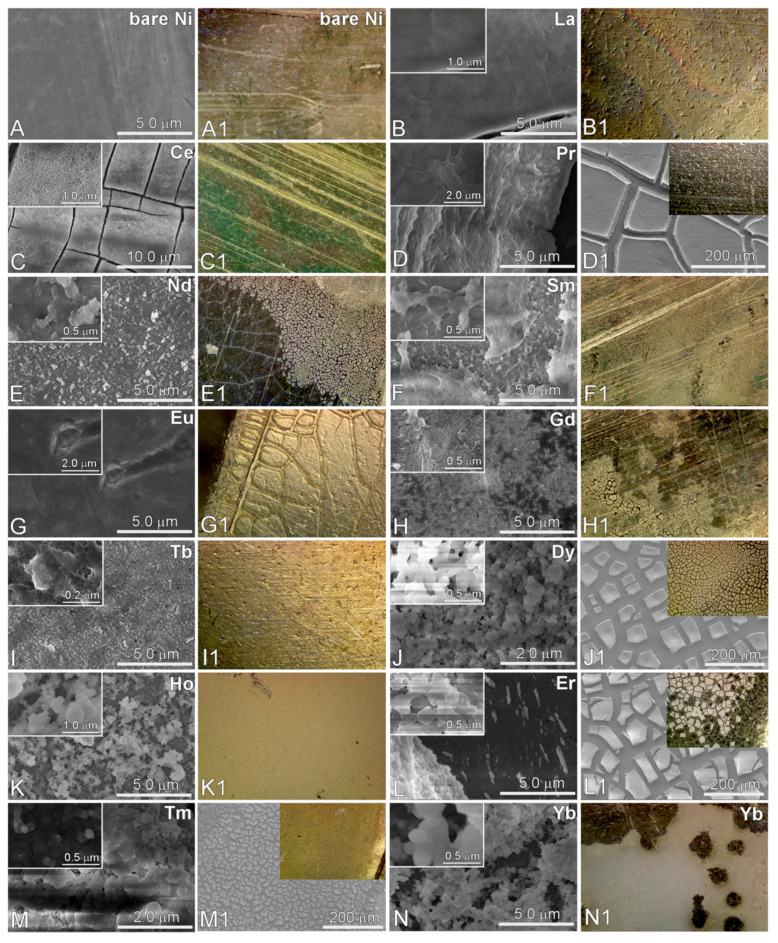
SEM images (**A**–**N**) and optical microscope (300×) images (**A1**–**N1**) are shown for a bare Ni sheet (**A**) and electrodeposited RE elements on a Ni sheet (from **B**–**N**).

To examine the electrochemical recovery of RE(III) ions over Ni sheets and the corresponding morphology, Figure 2 shows scanning electron microscope (SEM) and optical microscope images before and after electrodeposition. On the basis of the images, all the RE elements were efficiently deposited on Ni sheets. The deposited areas were clearly discriminated from the undeposited areas by optical microscope images. For the SEM images of La, Ce, and Pr, the surface morphology showed a uniform film state, although some cracks were present in the deposited areas. For the SEM images of Nd, Sm, Eu, Gd, and Tb, the morphology appeared to be a film state aggregated by ultrafine particles. For the SEM images of Dy, Ho, Er, Tm, and Yb, the particle sizes were appeared to be much bigger and become nanoparticle structures. Overall, it can be concluded that the morphology was changed from uniform thin film state to nanoparticle structures as the atomic number was increased. The present data can provide valuable information on the development of thin film fabrication by electrodeposition.

**Figure 3:**
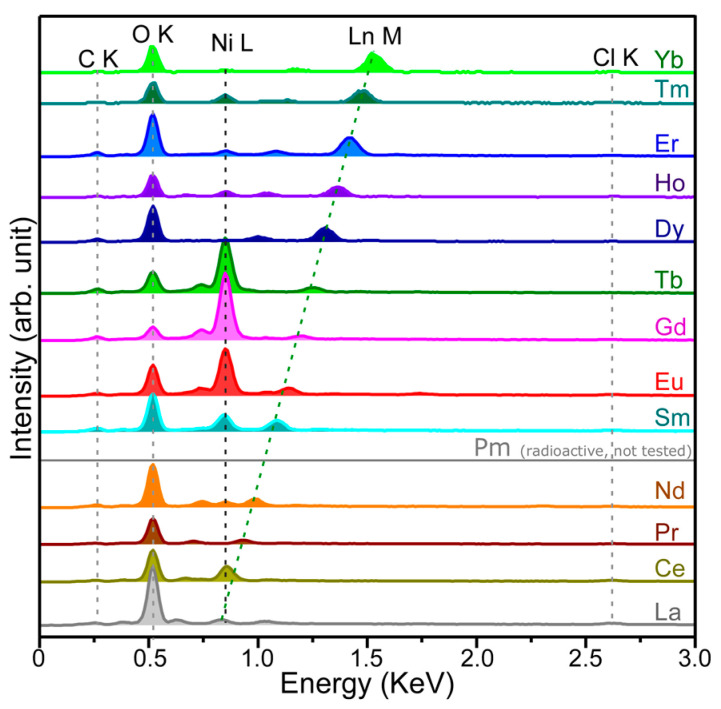
Energy-dispersive X-ray spectroscopy (EDXS) data for electrodeposited RE elements over a Ni sheet.

To confirm the elements of the recovered RE elements, Figure 3 shows the energy-dispersive X-ray spectroscopy (EDXS) data for the samples shown in Figure 2. For the EDX spectrum (not shown here) of a bare Ni sheet, the detected signals were of Ni (major), C (minor), and O (minor) elements: Ni L (0.84 KeV), C K (0.26 KeV), and O K (0.52 KeV) [24]. Upon electrodeposition, the signals of RE, C, and O were strongly increased. The strong O signal was plausibly due to RE–O, –OH, –ClO_4_, and –CO_3_ species, confirmed by Fourier transform infrared spectroscopy (FT-IR) and X-ray photoelectron spectroscopy (XPS) shown below. The EDXS signals of RE and Cl (Cl K = 2.62 KeV) elements were newly appeared. The Cl K signal was due to ClO_4_ species, further discussed in the FT-IR spectra below. The Ni L signals were observed to be dependent on the amounts of RE deposited on a Ni sheet. The Gd/Ni sample showed the strongest Ni signal while Dy/Ni showed the weakest signal. This indicates that Dy element was the most recovered by electrodeposition after a given time for 5 min. For the EDXS signals (Ln M) of RE elements, the peak position was linearly increased to a higher energy position with increasing the atomic number. The La M (#57 for La element) and the Yb M (#70 for Yb element) signals were positioned at 0.83 and 1.52 KeV, respectively [24]. The peak separation between the two peaks was estimated to be 0.69 KeV. For the radioactive promethium, Pm M can be expected to be observed around 1.1 KeV. On the basis of inductively coupled plasma elemental analysis of Ce and the Ln M EDXS signal intensities, the recovery percentages (%) were roughly estimated to be La (15.6%), Ce (3%), Pr (3.2%), Nd (5.7%), Sm (5.9%), Eu (4.3%), Gd (2.7%), Tb (3.3%), Dy (12.6%), Ho (11.5%), Er (12.7%), Tm (15.3%), and Yb (18.7%) over a 5 mm × 10 mm size electrode in 5 min.

**Figure 4:**
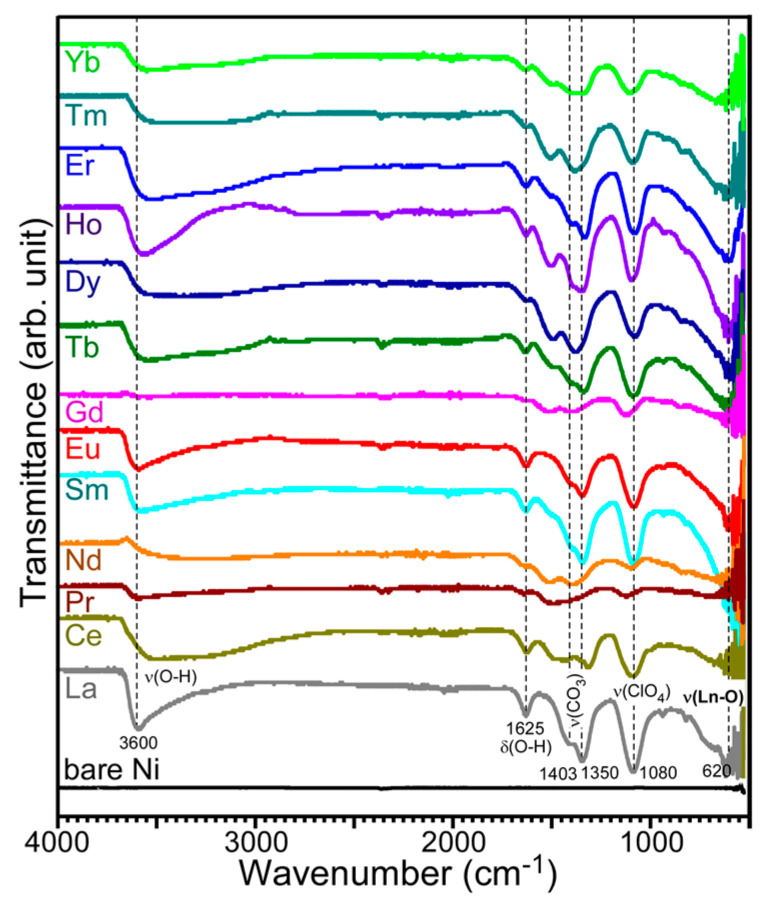
Transmittance FT-IR spectra for electrodeposited RE elements over a Ni sheet.

To deduce the chemical structures of the electrodeposited materials, Figure 4 displays the FT-IR spectra for all the electrodeposited samples. Very interestingly, all the FT-IR spectra were found to be similar, except for the signal intensity, which was determined by the amounts of recovered materials. The FT-IR intensity was in good consistent with the EDXS signal intensity. The Gd/Ni sample showed the weakest IR intensity. As discussed above, this sample showed the strongest EDXS Ni signal (from the Ni support). This indicates that the electrodeposited Gd was the thinnest. In the FT-IR spectra, a broad peak around 3600 cm^−1^ was distinctly observed, attributed to RE–OH stretching vibrations [25,26,27,28]. The corresponding O–H bending vibrational mode was observed at 1625 cm^−1^. The commonly appeared peak at 620 cm^−1^ was attributed to a RE–O vibrational mode [26]. A broad peak at 1080 cm^−1^ could be related with a vibration of ClO_4_^−^ group [29]. Two strong peaks were also observed at 1350 and 1403 cm^−1^, the most plausibly attributed to the stretching vibration modes of CO_3_^2−^ group [25,26,27,28]. On the basis of the FT-IR data, it could be concluded that the electrodeposited samples were complexes of RE, OH (and/or H_2_O), CO_3_^2−^, and ClO_4_^−^ groups, further discussed below.

**Figure 5:**
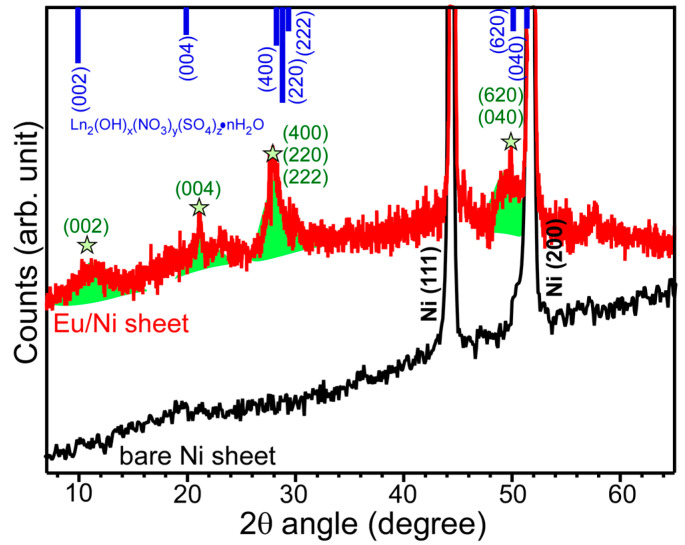
XRD data for bare Ni and selected electrodeposited Eu on a Ni sheet. The literature XRD patterns for Ln_2_(OH)*_x_*(NO_3_)*_y_*(SO_4_)*_z_*·nH_2_O complex are shown for comparison.

To further examine the crystal structures of the electrodeposited samples, the X-ray diffraction (XRD) patterns of a selected Eu sample and a bare Ni were displayed in Figure 5. The XRD signals were barely detected because the film was ultrathin (and low crystallinity), and the electrode was too small to be properly aligned. This needs further investigation to increase a potential applicability. For bare Ni sheet, the XRD signals were very strong and there were no significant impurity signals. The strong peaks at 2θ = 45° and 52° were attributed to the (111) and (200) crystal planes of metallic Ni [30]. The other (but clearly discriminated from the background signal) weak XRD signals were observed around 2θ = 10°, 20°, 28°, and 50°. These XRD peak positions are similar to those of Ln_2_(OH)*_x_*(NO_3_)*_y_*(SO_4_)*_z_*·nH_2_O complex [25] and metal carbonate hydroxide structures [25,27,28,31,32]. Assuming that anions of SO_4_^2−^ and NO_3_^−^ are exchanged by CO_3_^2−^ and ClO_4_^−^ but the crystal phase is not changed [25], the corresponding crystal planes are assigned on the four broad peaks in Figure 5. Based on the present XRD information, the FT-IR data, and the literature information, the recovered RE elements were proposed to be presented as RE_2_(OH)*_x_*(CO_3_)*_y_*_−_*_z_*(ClO_4_)*_z_*·nH_2_O complex, where O and RE were the most abundantly present in the complex [25]. The EDXS data commonly showed RE, C, O, and Cl elements, and the atomic compositions of RE and O were much higher than those of C and Cl.

**Figure 6:**
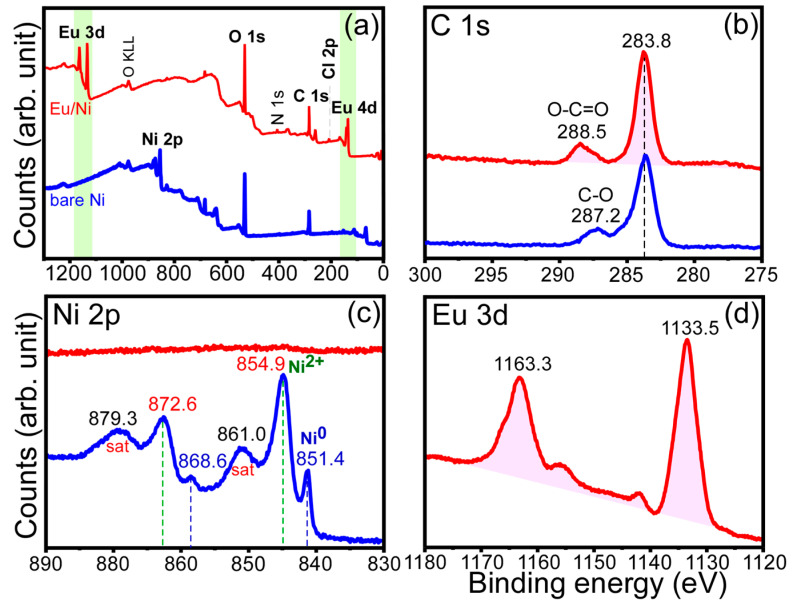
Survey (**a**), high-resolution C 1s (**b**), Ni 2p (**c**), and Eu 3d (**d**) XPS spectra before and after Eu electrodeposition over a bare Ni sheet.

Surface chemical states of a selected electrodeposited sample were examined by X-ray photoelectron spectroscopy (XPS). Figure 6 displays survey, high-resolution C 1s, Ni 2p, and Eu 3d XPS spectra before and after Eu electrodeposition over a Ni sheet. All the binding energies (BEs) here were not calibrated using an internal standard, but the XPS spectrometer showed an Au 4f_7/2_ XPS peak at 84.0 eV for a cleaned Au film (as an external standard). For the survey XPS scan of a bare Ni sheet, Ni, C and O elements were only observed. An O 1s XPS signal was observed at a BE of 530.2 eV for a bare Ni sheet, due to oxidation of Ni surface. The surface oxide was ultrathin and could not be detected by the bulk XRD technique, shown above. For the survey scan of an electrodeposited Eu on Ni, Eu 3d and Eu 4d signals were strongly and newly appeared. In addition, N 1s and Cl 2p XPS signals were also newly appeared. The N 1s peak position was observed at a BE of 406.0 eV [33,34], plausibly due to nitrates trapped in the surface. The Cl 2p BE was observed at 207.0 eV, due to ClO_4_ species [33,35]. The Cl element was also detected by EDXS, shown above. In the C 1s XPS for bare Ni, two peaks were observed at 283.8 eV (major) and 287.2 eV (minor), attributed to adventitious C–C and C–O species, respectively. In the C 1s XPS for electrodeposited Eu on Ni, a C 1 XPS peak at a BE of 288.5 eV was newly observed, attributed to O–C=O species such as carbonates [33]. In the Ni 2p XPS for bare Ni, several peaks were observed. Two shaper peaks at 868.6 and 851.4 eV with a spin orbit splitting of 17.2 eV were assigned to the Ni 2p_1/2_ and Ni 2p_3/2_ XPS signals of metallic Ni [30,33,36], respectively. Two broader peaks at 872.6 and 854.6 eV with a spin orbit splitting of 17.7 eV were assigned to the Ni 2p_1/2_ and Ni 2p_3/2_ XPS signals of Ni(II) state (e.g., NiO), respectively [30,36]. The corresponding satellite peaks of Ni(II) oxidation state were observed at 879.3 and 861.0 eV, respectively [33,36]. On the basis of the Ni 2p XPS, the surface of Ni sheet was oxidized to Ni(II) state. Upon Eu deposition, the Ni 2p signal was not observed, an indication that the electrodeposited overlayer was much thicker than the XPS probe depth. In the Eu 3d XPS, two major signals were observed at 1163.3 and 1133.5 eV with a spin-orbit splitting energy of 29.8 eV, attributed to Eu 3d_3/2_ and Eu 3d_5/2_ XPS signals of Eu(III) state, respectively [37]. A weaker Eu 3d_3/2_ peak around 1156.0 eV could be due to some Eu(II) states [37].

**Figure 7:**
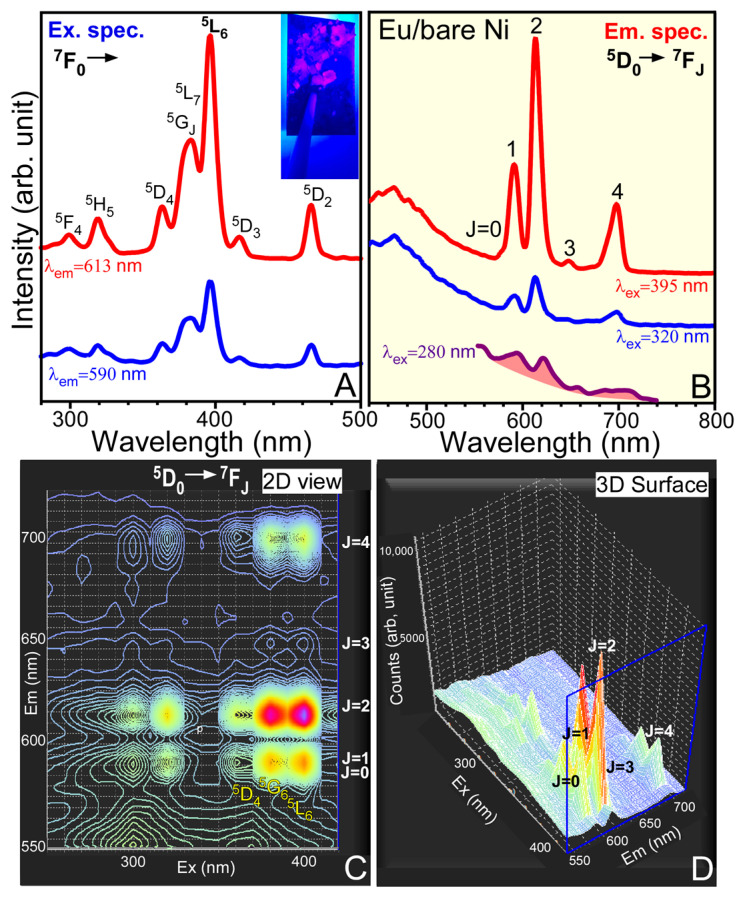
Excitation (**A**) and emission (**B**) spectra for a thick electrodeposited Eu on a Ni sheet, and the corresponding 2D- and 3D-PL contour mapping images (**C**, **D**).

To further confirm the oxidation state of Eu elements, photoluminescence spectroscopy was employed for the Eu electrodeposited sample because Eu(III) ion has unique ^5^D_0_ → ^7^F_0,1,2,3,4_ transitions in the visible region between 550 and 720 nm [37,38,39,40], while Eu(II) shows no visible light emission under an excitation of UV light. Figure 7 shows excitation and emission spectra for a thick electrodeposited Eu sample and the corresponding 2D- and 3D-PL contour mapping images. For the excitation spectra setting at emission wavelengths (λ_em_) of 613 and 590 nm, the PL profiles were found to be very similar, but the intensity was stronger for the spectra at λ_em_ = 613 nm. This indicates that the emission under 613 nm light was stronger than the emission under 590 nm light. Various peaks were observed at 299, 319, 363, 383, 395, 416, and 466 nm, assigned to the ^5^F_4_, ^5^H_5_, ^5^D_4_, ^5^G_J_/^5^L_7_, ^5^L_6_, ^5^D_3_, and ^5^D_2_ transitions from the ground ^7^F_0_ state of Eu(III), respectively [37]. For the emission spectra taken at excitation wavelengths (λ_ex_) of 280 nm (indirect excitation), 320 nm (the ^5^F_4_ ← ^7^F_0_ direct excitation), and 395 nm (the ^5^L_6_ ← ^7^F_0_ direct excitation), the two emission profiles at direct excitations of λ_ex_ = 320 and 395 nm are similar, but the emission profile at an indirect excitation of λ_ex_ = 280 nm is dissimilar to the other two. The PL intensity of the direct transition to an Eu(III) excited energy level was found to be stronger than that of the indirect transition. Several PL peak positions were commonly observed at 578, 590, 613, 647, and 698 nm, commonly associated to the ^5^D_0_ → ^7^F_0_, ^5^D_0_ → ^7^F_1_, ^5^D_0_ → ^7^F_2_, ^5^D_0_ → ^7^F_3_, and ^5^D_0_ → ^7^F_4_, transitions, respectively [37,38,39,40]. The ^5^D_0_ → ^7^F_2_ transition was observed to be the most intense. Because of the strong emission, the sample appeared to be red for the electrodeposited Eu (inset photo in Figure 7A). The ^5^D_0_ → ^7^F_2_ transition is known to be electric dipole transition and hypersensitive to Eu(III) local environment. The transition becomes dominant when Eu(III) ion is located at an asymmetric site. On the other hand, the ^5^D_0_ → ^7^F_1_ transition is insensitive to the local environment. For this reason, (^5^D_0_ → ^7^F_2_)/(^5^D_0_ → ^7^F_1_) intensity ratio is regarded as an asymmetric ratio for Eu(III) location site. The ratio was estimated to be 2.3 for the emission spectra at direction excitations. The ratio was 1.2 for the emission at indirect excitation. The corresponding 2D and 3D contour PL mapping profiles show densely spaced regions, an indication of strong emission signals. The densely spaced regions are mainly localized at the upper energy levels (^5^F_4_, ^5^H_5_, ^5^D_4_, ^5^G_J_/^5^L_7_, ^5^L_6_, ^5^D_3_, and ^5^D_2_) ← ^7^F_0_ excitation transitions and ^5^D_0_ → ^7^F_0_,_1,2,3,4_ emission transitions.

## 3. Experimental Section

### 3.1. Sample Preparations

Ni sheets (99.96%) were cut with a size of 5 mm × 20 mm; cleaned by ultrasonication in acetone, isopropyl alcohol, and water, repeatedly; and dried under an infrared lamp. All the purchased RE(III) ions were nitrate forms and used as received without any further purification: La(III) nitrate hexahydrate (99.999%, Sigma-Aldrich, Saint Louis, MO, USA), Ce(III) nitrate hexahydrate (99%, Sigma-Aldrich), Pr(III) nitrate pentahydrate (99.9%, Alfa Aesar, Ward Hill, MA, USA), Nd(III) nitrate hexahydrate (99.9%, Alfa Aesar), Sm(III) nitrate hexahydrate (99.9%, Alfa Aesar), Eu(III) nitrate hexahydrate (99.9%, Alfa Aesar), Gd(III) nitrate hexahydrate (99.9%, Sigma-Aldrich), Tb(III) nitrate hydrate (99.9%, Alfa Aesar), Dy(III) nitrate pentahydrate (99.9%, Sigma-Aldrich), Ho(III) nitrate pentahydrate (99.99%, Alfa Aesar), Er(III) nitrate hydrate (99.9%, Sigma-Aldrich), Tm(III) nitrate hydrate (99.9%, Alfa Aesar), and Yb(III) nitrate hydrate (99.9%, Sigma Aldrich). Sodium perchlorate (NaClO_4_ ≥ 98.0%, Sigma-Aldrich) was used as received and made to an aqueous 0.1 M solution as a supporting electrolyte. RE(III) ions were 10 mM concentration in the supporting electrolyte. Cyclic voltammetry tests were conducted using a WPG100 Potentiostat/Galvanostat (WonATech Co., Ltd., Seoul, Korea) electrochemical workstation. with a conventional three-electrode arrangement: a Ag/AgCl (3.5 M KCl) reference electrode, a Pt wire (0.5 mm) counter electrode, and a Ni sheet electrode (5 mm × 20 mm) in a 0.1 M NaClO_4_ electrolyte with and without RE(III) ions (10 mM). For the recovery of the RE elements, the electrodeposition over a Ni sheet was performed by amperometry for 5 min setting at the reduction potential observed in the CV data. After the electrodeposition, the electrode was gently washed with deionized water and dried under an infrared lamp before further characterization.

### 3.2. Characterization of the RE Recovered Materials

The morphology of the RE-recovered samples was examined using a field-emission Hitachi S-4800 SEM (FE-SEM, Hitach Ltd., Tokyo, Japan). The crystal phases of the electrodeposited sample were identified using a MiniFlex II X-ray diffractometer (Rigaku Corp., Tokyo, Japan) equipped with a Cu K_α_ radiation source. Energy-dispersive X-ray spectroscopy (EDXS) was performed using a JSM 7000 F scanning electron microscope (JEOL Ltd., Tokyo, Japan) at an acceleration voltage of 20 kV. The FT-IR spectra were recorded using a Nicolet iS 10 FT-IR spectrometer (Thermo Scientific Korea, Seoul, Korea) with an attenuated total reflection mode. Photoluminescence (PL) emission and excitation spectra were obtained using a Sinco FS-2 fluorescence spectrometer (Sinco, Seoul, Korea) setting at scan speed = 300 nm/min, slit width = 5 nm, PMT voltage = 700 V, and integration time = 20 ms. The emission profiles were recorded at various excitation wavelengths to plot 2D/3D PL contour profiles. X-ray photoelectron spectroscopy was employed using Thermo-VG Scientific K-alpha^+^ spectrometer (Thermo VG Scientific, Waltham, MA, USA) with a monochromatic Al *K*_α_ X-ray source (spot size = 400 μm) and a hemispherical energy analyzer (CAE mode, pass energy = 50.0 eV, and step size = 0.100 eV). An Avio 500 inductively coupled plasma optical emission spectrometer (Perkin Elmer, Waltham, MA, USA) was used to examine the amount of Ce ions present in the electrolyte after the amperometry test for 5 min.

## 4. Conclusions

In this work, we first showed a big data set for the recovery of rare earth (La, Ce, Pr, Nd, Sm, Eu, Gd, Tb, Dy, Ho, Er, Tm, and Yb) ions over a Ni sheet in a 0.1 M NaClO_4_ electrolyte. Cyclic voltammetry tests showed the reduction potentials between −1.2 and −0.8 V (vs. Ag/AgCl). The observed reduction potentials were reported for all the RE elements with increasing the atomic number. SEM images showed that the electrodeposition method was useful to recover the RE elements as thin film or nanoparticle structures on a Ni sheet. As the atomic number was increased the morphology was changed from uniform thin film state to nanoparticle structures. The electrodeposited La (#59) on Ni showed a uniform film morphology while the electrodeposited Yb (#70) on a Ni sheet showed nanoparticle morphology. EDXS data confirmed the elements of RE, C, O, and Cl. The EDXS Ni L signal was used to estimate the relative amounts of electrodeposited RE materials. On the basis of the FT-IR and XRD data, it was tentatively concluded that the electrodeposited recovered materials were of a RE_2_(OH)*_x_*(CO_3_)*_y_*_−*z*_(ClO_4_)*_z_*·nH_2_O complex. For a selected electrodeposited Eu on Ni, the oxidation state of 3+ was confirmed by Eu 3d XPS signals. The Eu(III) oxidation state was further confirmed by the PL spectra showing the ^5^D_0_ → ^7^F_0_,_1,2,3,4_ emission transitions in the visible region between 570 and 720 nm. Future potential experiments may include a clear elucidation of the crystal structure, and electrodeposition over a porous Ni mesh for energy storage and energy production (e.g., CO_2_ reduction and water splitting) electrochemical electrodes.

Overall, the present study contains very valuable electrochemical behaviors of all the RE(III) ions in a NaClO_4_ electrolyte when a Ni sheet is used as a working electrode. The newly established big data set for RE elements could be used to predict the redox behaviors of Ln and An elements present in solutions. Furthermore, their morphologies, chemical states and the electrochemical recovery method could provide valuable information on the reprocessing of used nuclear oxide fuels, the recovery of RE-containing industry wastes, and fabrication of thin films.
